# Correlation Between Cognition and Balance Among Middle-Aged and Older Adults Observed Through a Tai Chi Intervention Program

**DOI:** 10.3389/fpsyg.2020.00668

**Published:** 2020-04-06

**Authors:** Tao Xiao, Lin Yang, Lee Smith, Paul D. Loprinzi, Nicola Veronese, Jie Yao, Zonghao Zhang, Jane Jie Yu

**Affiliations:** ^1^College of Mathematics and Statistics, Shenzhen University, Shenzhen, China; ^2^Cancer Epidemiology and Prevention Research, Alberta Health Services, Calgary, AB, Canada; ^3^Departments of Oncology and Community Health Sciences, Cumming School of Medicine, University of Calgary, Calgary, AB, Canada; ^4^Cambridge Centre for Sport and Exercise Sciences, Anglia Ruskin University, Cambridge, United Kingdom; ^5^Department of Health, Exercise Science and Recreation Management School of Applied Sciences, The University of Mississippi, Oxford, MS, United States; ^6^Geriatrics Division, Department of Medicine–DIMED, University of Padova, Padua, Italy; ^7^School of Humanities and Social Sciences, Harbin Institute of Technology (Shenzhen), Shenzhen, China; ^8^College of Physical Education, Soochow University, Suzhou, China; ^9^Department of Sports Science and Physical Education, The Chinese University of Hong Kong, Hong Kong, China

**Keywords:** cognition, alternative exercise, postural control, equilibrium, Tai Chi

## Abstract

**Background:**

Age-associated decline in cognition and balance may cause severe ability loss for daily living activities among middle-aged and older adults. The relationship between cognition and balance in this aging population remains to be explored.

**Objective:**

The present study Is exploratory in nature and aimed to examine the relationship between balance (both static and dynamic components) and global cognitive function among middle-aged and older adults through Tai Chi (TC) practice as a research avenue.

**Methods:**

A short-term (12 weeks) intervention of TC was conducted among middle-aged and older adults in the community setting. Global cognitive function (using the Chinese version of the Montreal Cognitive Assessment score (MoCA) and balance (i.e., one leg standing test score; Timed Up and Go Test score, TUGT) of all participants were assessed before and after the intervention. Age, body mass index (BMI), sex, and physical fitness variables (Chair Stand Test, CST; the 6-Meter Walk Test, 6MWT) were also collected as confounding factors.

**Results:**

Significant moderator effects of baseline CST on the association between the dichotomized baseline MoCA score and the baseline left leg balance score (*p* = 0.0247), the baseline right leg balance score (*p* = 0.0140) and the baseline TUGT score (*p* = 0.0346) were found. Change score of left score balance (*p* = 0.0192) and change score of TUGT (*p* = 0.0162) were found to be significantly associated with change score of cognitive function.

**Conclusion:**

Cognitive function and balance are interrelated in middle-aged and older adults. The association between global cognitive function and balance Is moderated by strength of lower limbs. The change scores of cognitive function and balance introduced by TC training were found to be positively correlated. Future research Is warranted to further confirm the cause-effect relationship of cognitive function and balance and its influencing factors among middle-aged and older adults utilizing intervention studies with larger sample sizes.

## Introduction

A large proportion of older adults live alone or are without children of any age at home. For example, in China, half (49.7%) of those 60 years and older live with no children, and this proportion increases to 56.1% in large and medium-sized cities (China Population Press, 2015). An increasing number of older adults living alone or without children exerts financial pressure on families and society as a whole (Asian Development Bank, 2014; Whetten-Goldstein et al., 2015). Moreover, greater financial pressures are more likely when older adults lose independence in activities of daily living such as walking, feeding, dressing and grooming, toileting, bathing, transferring, cooking, transportation, and shopping, etc. (Mlinac and Feng, 2016). Fall injuries and dementia are the most frequent and costly age-induced factors that may cause complete loss of independence in activities of daily living. According to the Centers for Disease Control and Prevention, falling Is the leading cause of fatal and non-fatal injuries among older adults, and more than one fourth of adults aged 65 years or above will fall each year (CDC Newsroom, 2016). For instance, among older Americans, around 29 million falls occur each year, resulting in 3 million emergency department visits, 800,000 hospitalizations, and 28,000 deaths. The average hospital cost of a fall encounter Is over US$30,000 and the annual Medicare costs for falls for individuals residing in the US aged 65 years and above was ∼US$31 billion in the year 2015 (Laurence and Michel, 2017). According to the World Health Organization, around 50 million individuals, globally, are currently living with dementia and nearly 10 million new cases occur each year (World Health Organization [WHO], 2017). The consequences incurred by decline in cognition are as serious as those by falls among older people. Thus far, there has not been a curative treatment for dementia (Koller et al., 2016). Hence, it Is important to maintain or slow down the degrading processes of both balance and cognitive function among older adults and even at an earlier age stage (i.e., middle-aged adults) to reduce the risks of falling (Iwasaki and Yamasoba, 2015; Zou et al., 2017b, 2018a) and dementia (Morris, 2012; Petersen, 2016). A large 10-years study of middle-aged to older adults (45–70) found that cognitive decline begins in the 45–55 decade (Singh-Manoux et al., 2012). The central ability to inhibit balance destabilizing vision-related postural control processes was found among middle-aged and older adults to depend at least partially on striatal dopaminergic pathways (Cham et al., 2007). The relationship between cognitive function and balance among middle-aged and older adults calls for potentially effective interventions that might improve both cognition and balance for this population.

Physical activity or exercise (one domain of physical activity) intervention programs have been shown to delay age-related balance or cognitive decline (Chase et al., 2016; Zou et al., 2017a; Gheysen et al., 2018; Zhang et al., 2018). Tai Chi (TC) has been recognized to promote both fall-related balance ability and cognitive function in the aging population (Wayne et al., 2014; Huang et al., 2017). TC Is a mind-body exercise with special features that have shown to be effective for improving balance and cognition together. Transitions between double- and single-leg stance commonly occur throughout entire TC routines (Wayne et al., 2016) which can potentially strengthen lower-limb muscle and flexibility, improving postural control. Additionally, TC consists of relatively complex movement sequences, which may benefit practitioners with memory training opportunities. To perform such sequences smoothly and connectedly, higher-order cognition (e.g., visuospatial ability, orientation, attentional resource allocation, and executive function) Is needed. Thus, TC may help facilitate greater conscious control over the entire body. Such practice may lead to a combined promotion in cognitive function and balance over time (Liu et al., 2018, 2019; Zou et al., 2018b,c,d, 2019a; Kong et al., 2019). Therefore, TC training may be a potential research avenue for investigating the relationship of cognitive function and balance. However, previous studies that investigated the intervention effects of TC on age-related decline in cognition and balance, and that evaluated both outcomes simultaneously are limited. Recently, several large-scale epidemiological studies showed that balance and cognition are highly interdependent (Clouston et al., 2013; Smith-Ray et al., 2015; Demnitz et al., 2016). Synergistic effects on improvements in both balance control and cognitive functioning were found by exercises combining cognitive and balance training, including multimodal exercise (Marmeleira et al., 2018), unicycling training (Weber et al., 2019), and mind-body exercise like yoga (Subramaniam and Bhatt, 2017). In addition, cognitive functions were also reported to be highly associated with mobility among middle-aged and older adults (Buchman et al., 2011; Demnitz et al., 2016, 2018; Li et al., 2018). The authors of the present study conducted a randomized trial, with modified Chen-style TC as case and 24-style active TC as control, to verify the effectiveness of TC training on improving cognitive function and balance (Zou et al., 2019b). By re-exploring and pooling the data from this randomized trial, the main purpose of this study was to examine the relationship between balance (both static and dynamic components) and global cognitive function under a condition of TC training among middle-aged and older adults controlling for key covariates (e.g., age, sex, mobility level). It Is hypothesized that there would be a positive and interdependent relationship between global cognitive function and balance among middle-aged and older adults.

## Methods

### Experimental Design

A 12-week intervention of TC was conducted among middle-aged and older adults. Baseline and posttest data on balance and cognitive function were collected; baseline and posttest data on confounding factors of fitness of lower limbs and aerobic exercise capacity were also collected; data on other confounding factors (i.e., age, gender, height, weight, and heart rate) were collected at baseline. Ethical approval was obtained from the research ethics committee before the commencement of this study.

### Study Participants

Participants included 78 Chinese middle-aged and older adults (mean age: 58.92 ± 8.03 years) and consisted of 37 male individuals and 41 female individuals. Participants were recruited from Yongzhou city of Hunan province in China between December 2017 and March 2018. Individuals who had no physical limitations in participating in TC practice and had not received any exercise training programs or practiced TC regularly over the past 3 months were included. Those individuals who reported serious diseases (e.g., cardiovascular disease, clinical depression) or a history of alcohol abuse, and who were not able to understand and follow the instructions in the pilot training session were excluded. All participants voluntarily participated in this study and provided written informed consent.

### Tai Chi Intervention Program

The intervention was a 12-week TC program consisting of two phases. All participants attended training for 1 h per session (including 10 min of warm-up, 40 min of TC practice, and 10 min of cool-down), 3 sessions per week for the first 6 weeks in a public park in the community of the city. The purpose of the first 6-week of the intervention was to guarantee that all participants understood the instructions and mastered the essentials of TC movements. The duration and frequency of practice were progressively increased during the last 6 weeks. During the latter half of the intervention, participants trained for one, and a half hours (including 10 min of warm-up, 70 min TC practice, and 10 min cool-down) in each session, five sessions per week for 6 weeks. In total, all participants practiced TC for 2,820 min over 12 weeks in a group format (∼40 participants per class). The postures involved in TC practice were from two different TC styles (modified Chen-style and 24-style) with different difficulty levels. The intervention was led by one experienced TC instructor with more than 15 years of TC experience. The comparisons of the intervention effects between these two TC styles have been reported elsewhere (Zou et al., 2019b). The present study focuses on investigating correlations between improvements of cognition and balance through the TC intervention program and therefore the two TC groups were pooled together.

### Outcome Measures

#### Global Cognitive Function

Global cognitive function was assessed using the Bejing version Montreal Cognitive Assessment (MoCA)^1^ that has been used in Chinese in previous research (Lu et al., 2011). The MoCA Is a validated brief paper-and-pencil screening test for cognitive function and detecting cognitive impairments. It consists of 12 elements to assess a variety of cognitive domains, including visuospatial ability, attention, executive function, language, orientation, memory, and abstraction. The total score of MoCA ranges from 0 to 30, with high scores representing high global cognitive ability and a score of <26 considered as experiencing symptoms of cognitive impairments.

#### Balance

The one leg standing test was used to evaluate static balance in unilateral stance. With their eyes closed, the participants were instructed to stand on the leg they selected, lift the knee of the other leg to ∼90°, keep their arms by their sides, and maintain balance without any assistive device. Both legs were tested successively with three attempts for each leg. To prevent falls or injuries, the tester stood close to the participants throughout the trials. The test was over when either the stance foot shifted or when the lifted foot was placed on the ground. The stance duration (in seconds) for each leg in each attempt was recorded using a stopwatch and the best (longest) score was chosen and included for data analyses (Michikawa et al., 2009).

The Timed Up and Go Test (TUGT), which Is widely used in elderly populations, was used to measure dynamic balance. The TUGT measures the time taken by an individual to rise from a standard chair (with the height of ∼45 cm), walk three meters, turn around, walk back to the chair, and sit down without any assistive device throughout. The participants were asked to be seated on the chair with their arms resting on the chair’s arms, and then stand up on the word “go” command. The recording time started on the “go” command and stopped as the participant sat down (Chan et al., 2017). Each participant had three attempts with the best (fastest) attempt used in the analysis.

#### Confounding Variables

Each participant provided demographic information in a survey, including age and sex. Participants’ heart rate (HR), body height and weight (Healthometer 402 KL Beam Scale weight/Height Rod, PELSTAR, McCook, IL, United States) were assessed at baseline. Body mass index (BMI; kg/m^2^) was calculated. The performance of balance has been widely reported as closely related to individuals’ physical mobility and fitness level (Nakano et al., 2014). Chair Stand Test (CST) and the 6-Meter Walk Test (6MWT) were used to assess the strength of lower limbs and aerobic endurance, respectively, at baseline and immediately after the intervention (12 weeks), and therefore considered as confounding factors in this study regarding the relation between cognition and balance. These two tests have been used in the elderly population, with further details discussed elsewhere (Zhou et al., 2019).

### Statistical Analysis

All statistical analyses were carried out using R (version 3.6.1) (R Core Team, 2019). First, associations between the baseline score of the cognitive parameter MoCA and the baseline score of each of the three balance parameters, namely, left leg balance, right leg balance, and TUGT were assessed using regression analysis to control for potential confounders, with interaction effects between balance parameters and confounding variables considered. As the baseline MoCA scores in the present data were not normally distributed (Shapiro–Wilk Normality Test statistic W = 0.94, *p* = 0.0007), they were dichotomized at a recommended clinical cutoff of 26 (coded as 1 or normal: MoCA score ≥26; coded as 0 or cognitive impairment: MoCA score < 26) (Hu et al., 2013). The varImp (abbreviation for “variance importance”) function of the caret package (version 6.0–84) in R was used to report the relative raw importance of all the available factors in the data that are potentially correlated to the dichotomized baseline MoCA, based on main-effect-only logistic regression model (Gevrey et al., 2003). Then three logistic regression analyses were used, with the dependent variable being the dichotomized baseline MoCA score, and independent variables being each of the three baseline balance parameters (i.e., Left Leg Balance score, Right Leg Balance score, or TUGT score) respectively, plus all of the six confounding variables (i.e., Gender, Age, HR, BMI, CST, and 6MWT), as well as their interaction terms with the corresponding baseline balance parameter. Second, four paired-sample t-tests were carried out to assess the change score volume of MoCA, left leg balance, right leg balance, and TUGT across time. Third, associations between the change score of MoCA and the change score of each of the three balance parameters were assessed by three linear regression models, controlling for change score of CST, change score of 6MWT, Gender, Age, HR, and BMI.

## Results

Seventeen out of the total 78 participants in this study were classified as having cognitive impairment (i.e., with MoCA score <26). The characteristics of all participants and descriptive information of confounding variables are presented in Table 1. Mean and standard deviation (M ± SD) of the raw data on all outcome measures at baseline and post-intervention, as well as the corresponding paired t-test results for these pre and post intervention data are presented in Table 2. All parameters were improved after the TC intervention. No participant had MoCA score less than 26 after the TC intervention. As noted earlier, these paired *t*-test results of significant improvements of cognitive function and balance introduced by TC intervention listed in Table 2 have already been confirmed with a different statistical method (i.e., the generalized estimation equations) for randomized trials on these data in a previously published paper (Zou et al., 2019b). Therefore, these significant results are not new but consistent findings with different type of test statistic.

**TABLE 1:** Confounder information of study participants at baseline and posttest (*N* = 78).

Variable	Distribution	

Gender	Baseline	Posttest
Male (*n*)	37	
Female (*n*)	41	
Age (years)	58.92 ± 8.03	
Heart Rate (HR)	74.51 ± 7.13	
Body mass index (BMI) (kg/m^2^)	24.61 ± 2.42	
Chair stand test (CST)	9.36 ± 2.14	8.39 ± 1.61
6-meter walk test (6MWT)	5.12 ± 0.99	4.93 ± 0.83

*Sample frequencies are displayed for each level of categorical variable; sample mean and standard deviation are displayed for each continuous variable as the form of “M ± SD”.*

**TABLE 2:** Cognitive function and balance data at baseline and posttest, and their paired *t*-test results.

Variable	Baseline	Posttest	Paired *t*-test statistic	*p*
Global cognitive function (MoCA)	26.41 ± 1.40	29.86 ± 0.45	22.81	< 2.2e–16 ^∗∗∗^
Static balance (left leg balance)	3.72 ± 2.54	5.63 ± 3.14	11.81	< 2.2e–16 ^∗∗∗^
Static balance (right leg balance)	3.61 ± 2.82	5.46 ± 2.92	9.86	2.62e–15^∗∗∗^
Dynamic balance (TUGT)	10.10 ± 2.47	8.95 ± 1.81	−9.01	1.16e–13^∗∗∗^

Sample mean and standard deviation are displayed for the data as the form of “M ± SD”; Significance code: ‘***’p < 0.001.

### *Raw* Relationships Between Cognitive Function *and* Balance Parameters *at* Baseline

Estimated logistic regression coefficients corresponding to the associations between the dichotomized baseline MoCA score and the baseline balance parameter scores controlling for other covariates with interaction terms are presented in Table 3. Similar significant tendencies were found for the correlations between the cognitive function and each of the three balance scores. A significant and positive (0.235) interaction effect between left leg balance and CST was found (95% CI 0.030–0.441, *p* = 0.0247) combined with the positively (3.287) estimated coefficient of the main effect of left leg balance score, this finding implies that high physical fitness Is associated with high positive correlation between the global cognitive function and the left leg balance. A significant and positive (11.922) coefficient estimate of the main effect of right leg balance score was found (95% CI 1.418–22.425, *p* = 0.00261), implying a significantly positive correlation between the global cognitive function and the right leg balance; a significant and positive (0.242) interaction effect between right leg balance and CST was also found (95% CI 0.049–0.435, *p* = 0.0140), implying that high physical fitness Is associated with high positive correlation between the global cognitive function and the left leg balance. A significant and negative (−0.274) interaction effect between TUGT and CST was found (95% CI −0.528 to −0.020, *p* = 0.0346) combined with the negatively (−4.806) estimated coefficient of the main effect of CST score, this finding implies that high physical fitness Is associated with high positive correlation between the global cognitive function and the dynamic balance (note that low TUGT score stands for high balance ability). These results indicate that the association between the baseline global cognitive function and the baseline static and dynamic balance abilities both increase with increased strength of lower limbs among middle-aged and older adults.

**TABLE 3:** Results of association analysis between the baseline MoCA and baseline balance scores.

Dependent variable			Dichotomized MoCA			

Independent variable	Left leg balance		Right leg balance		TUGT	
	Coef.	*p*	Coef.	*p*	Coef.	*p*
Balance	3.287	0.3639	11.922	0.0261^∗^	–4.806	0.1914
Balance*HR	0.001	0.9756	–0.062	0.1821	0.031	0.3857
Balance*Gender	0.528	0.2097	–0.250	0.5874	–0.635	0.1613
Balance*Age	0.002	0.9468	–0.078	0.1166	0.057	0.1769
Balance*BMI	–0.189	0.0686	–0.187	0.1484	0.017	0.8864
Balance*CST	0.235	0.0247^∗^	0.242	0.0140^∗^	–0.274	0.0346^∗^
Balance*6MWT	–0.252	0.1944	–0.130	0.6209	0.355	0.1213

MoCA; The Chinese version of the Montreal Cognitive Assessment; BMI, body mass index; CST, Chair Stand Test; TUGT, Timed Up and Go Test; Significance code: ‘*’p < 0.05.

### Relationship Between Improvements *in* Cognitive Function *and* Balance After TC Intervention

The associations between the change score of MoCA and the change score of each of the balance parameters are presented in Table 4. These associations are represented by regression coefficients and their p-values. As comparisons, we also provide association results between the change score of MoCA and the change scores of physical fitness parameters (CSTΔ and 6MWTΔ). The association between the change score of MoCA and the change score of left leg balance Is significantly positive with the corresponding regression coefficient estimated as 0.251 (95% CI 0.046–0.457, *p* = 0.0192). The association between the change score of MoCA and the change score of TUGT Is significantly positive with the corresponding regression coefficient estimated as 0.349 (95% CI 0.071–0.626, *p* = 0.0162). The change score of right leg balance, as well as the change scores of physical fitness parameters (CSTΔ and 6MWTΔ) do not present significant associations with change score of MoCA. These results indicate that the improvement of cognitive function introduced by TC training among middle-aged and older adults Is associated with change of left leg static balance and dynamic balance, but not with change of right leg static balance and physical fitness.

**TABLE 4:** Results of the association between the change scores of MoCA and those of the balance parameters.

Dependent Variable			MoCAΔ			

Independent Variable	Left leg balanceΔ		Right leg balanceΔ		TUGTΔ	
	Coef.	*p*	Coef.	*p*	Coef.	*p*
*BalanceΔ*	0.251	0.0192^∗^	–0.025	0.7985	0.349	0.0162^∗^
6MWTΔ	0.106	0.5693	0.069	0.7229	–0.050	0.7937
CSTΔ	–0.120	0.3866	–0.105	0.4812	–0.208	0.1507

Δ: change score; Significance code: ‘*’p < 0.05.

## Discussion

The primary purpose of this study was to investigate the dynamic relationship between cognitive function and balance among middle-aged and older adults through TC practice while considering influencing factors such as physical fitness (i.e., strength of lower limbs and aerobic endurance). Major findings of this study include: (1) cognitive function was related to static and dynamic balance, and such correlation increases with increased strength of lower limbs; (2) Improvements in cognitive function Is also potentially associated with improvements in left leg (usually non-dominant leg) static balance and dynamic balance, but not associated with improvements in right leg (usually dominant leg) balance and physical fitness introduced by TC training.

Cognitive function and balance remarkably decline with advancing age, which often restricts participation in activities of daily living and may contribute to secondary health risks (e.g., falls) in the elderly. In line with previous studies (Pichierri et al., 2011; Alonso et al., 2016), we found that cognitive function and balance are interrelated among middle-aged and older adults. This relationship can be explained by the competition of limited central attentional resources in maintaining posture and performing cognitive tasks during activities of daily living. Moreover, when compared to young individuals, middle-aged, and older adults usually experience more cognitive distractions and spend more attentional resources in generating complex movements due to the natural ageing process (Pichierri et al., 2011). Additionally, the results in this study demonstrated the importance of muscle strength as a moderator for the relationship between improvement of cognition and that of balance among middle-aged and older adults, which Is consistent with previous literature that muscle strength serves as an important role in performing activities requiring balance and cognitive functioning (Alonso et al., 2016), and that low levels of muscle training or physical activity are a risk factor for cognitive functioning or balance in middle-aged and older adults (Holviala et al., 2006; de Souto Barreto et al., 2016).

There Is a growing interest in merging physical and cognitive stimulation (mind-body exercise), such as TC and Yoga, in health promotion in aging individuals (Ni et al., 2014; Gothe et al., 2016; Marmeleira et al., 2018). Furthermore, a recent systematic review suggested that interventions incorporating both physical and cognitive components not only benefit cognitive function, but also physical functions, including balance (Booth et al., 2016). In the present study the association of cognitive function with balance through a TC intervention program Is comparable to a cross-sectional study (Leandri et al., 2015) that evaluated cognitive function using MoCA and static balance with participants eyes closed (similar to the present study). The study found a significant association between static and dynamic balance and cognitive function in middle-aged and older adults. These findings suggest that this relationship may due to the link between the vestibular system and the hippocampus in memory performance. Future research, using neuroimaging techniques, should investigate this in future.

The major strengths of this study include the assessment of both cognitive function and balance with the consideration of other key confounding factors and the advanced statistical analyses. Although this study was exploratory in nature, these findings will add to the current literature by improving our understanding of the relationship between cognitive function and balance and the mechanism behind the beneficial effects of TC on cognition. Such research will be valuable to professionals and practitioners who have concerns with the prevention and treatment of age-related deterioration in cognitive and physical functioning in the elderly.

Despite the above advantages, findings from this study must be interpreted in light of its limitations. First, MoCA evaluates global cognitive function as a diagnostic measurement, which may lack some degree of sensitivity to TC training. In addition, the dichotomization of the MoCA variable in the study may also limit its sensitivity to TC training, or perhaps over-emphasizes its effect. Future studies should focus on the specific components of cognitive function, such as executive function and memory. Second, all participants were Chinese, which limits the generalizability of our findings to other non-Chinese populations.

## Conclusion

Cognitive function and balance are interrelated in middle-aged and older adults when using TC practice as a new research avenue for this investigation. The global cognitive function Is associated with balance, and such association Is moderated by strength of lower limbs. Trainings or exercise forms designed for improving balance may be also likely facilitate cognitive function among middle-aged and older adults. Future research Is warranted to further confirm the cause-effect relationship of cognitive function and balance among middle-aged and older adults and its influencing factors in intervention studies with larger sample size.

## Data Availability Statement

The datasets generated for this study are available on request to the corresponding author.

## Ethics Statement

The studies involving human participants were reviewed and approved by Hunan University of Science and Technology College of Sport Science Research Ethics Committee. The patients/participants provided their written informed consent to participate in this study.

## Author Contributions

JY, TX, and ZZ: conceptualization. TX, JY, and LY: methodology. TX: formal analysis. TX, LY, LS, JY, PL, NV, ZZ, and JY: writing – original draft preparation and writing – review and editing.

## Conflict of Interest

The authors declare that the research was conducted in the absence of any commercial or financial relationships that could be construed as a potential conflict of interest.

## Footnotes

1 www.mocatest.org
